# Self-Monitoring Kidney Function Post Transplantation: Reliability of Patient-Reported Data

**DOI:** 10.2196/jmir.7542

**Published:** 2017-09-26

**Authors:** Céline van Lint, Wenxin Wang, Sandra van Dijk, Willem-Paul Brinkman, Ton JM Rövekamp, Mark A Neerincx, Ton J Rabelink, Paul JM van der Boog

**Affiliations:** ^1^ Leiden University Medical Center Department of Nephrology Leiden Netherlands; ^2^ Interactive Intelligence Group Delft University of Technology Delft Netherlands; ^3^ Department of Health, Medical and Neuropsychology and Behavioural Sciences Leiden University Leiden Netherlands; ^4^ Dutch Organization for Applied Scientific Research (TNO) the Hague Netherlands

**Keywords:** self-care, kidney transplantation, creatinine, patient compliance, data accuracy, patient reported outcomes

## Abstract

**Background:**

The high frequency of outpatient visits after kidney transplantation is burdensome to both the recovering patient and health care capacity. Self-monitoring kidney function offers a promising strategy to reduce the number of these outpatient visits.

**Objective:**

The objective of this study was to investigate whether it is safe to rely on patients’ self-measurements of creatinine and blood pressure, using data from a self-management randomized controlled trial.

**Methods:**

For self-monitoring creatinine, each participant received a StatSensor Xpress-i Creatinine Meter and related test material. For self-monitoring blood pressure, each participant received a Microlife WatchBP Home, an oscillometric device for blood pressure self-measurement on the upper arm. Both devices had a memory function and the option to download stored values to a computer. During the first year post transplantation, 54 patients registered their self-measured creatinine values in a Web-based Self-Management Support System (SMSS) which provided automatic feedback on the registered values (eg, seek contact with hospital). Values registered in the SMSS were compared with those logged automatically in the creatinine device to study reliability of registered data. Adherence to measurement frequency was determined by comparing the number of requested with the number of performed measurements. To study adherence to provided feedback, SMSS-logged feedback and information from the electronic hospital files were analyzed.

**Results:**

Level of adherence was highest during months 2-4 post transplantation with over 90% (42/47) of patients performing at least 75% of the requested measurements. Overall, 87.00% (3448/3963) of all registered creatinine values were entered correctly, although values were often registered several days later. If (the number of) measured and registered values deviated, the mean of registered creatinine values was significantly lower than what was measured, suggesting active selection of lower creatinine values. Adherence to SMSS feedback ranged from 53% (14/24) to 85% (33/39), depending on the specific feedback.

**Conclusions:**

Patients’ tendency to postpone registration and to select lower creatinine values for registration and the suboptimal adherence to the feedback provided by the SMSS might challenge safety. This should be well considered when designing self-monitoring care systems, for example by ensuring that self-measured data are transferred automatically to an SMSS.

## Introduction

After kidney transplantation, an early detection of transplant failure is mandatory to minimize permanent damage to the transplanted organ. For kidneys, blood level of creatinine is considered the most important indicator of kidney function [1]. Patients therefore have their serum creatinine checked on average 20 times during the first year post transplantation. As hypertension is both a potential indicator of decreased kidney function and an important risk factor for kidney graft failure [2-5], blood pressure needs extensive monitoring too. If patients were enabled to monitor both parameters at home, this would have important advantages. Self-monitoring could improve the speed of rejection detection as measurements can take place more frequently while at the same time the high number of outpatient visits could be reduced and replaced by telephonic consults. Furthermore, giving patients a more active role in their own care through self-monitoring has been shown to be of clinical benefit for a wide range of patients with chronic disease [6-15] and to lead to a higher quality of life [16-19]and more patient empowerment [7,19-22].

A pilot study of our own group showed that self-monitoring of both blood pressure and creatinine is very well accepted among patients, suggesting that at-home monitoring after transplantation offers a promising strategy [23]. For self-monitoring to be a safe alternative to regular face-to-face follow-up, however, patients need to adhere to a monitoring schedule, report test results accurately, and act upon test results if these suggest graft failure may occur. This is important for all patients who engage in self-monitoring, but especially for patients who are transplanted. As most patients who develop graft rejection are asymptomatic and present with an increased serum creatinine only, frequent measuring is essential to make the difference between treatment in time and damage to or even loss of the kidney transplant. Level of adherence to a self-monitoring schedule has been shown to vary widely in other disease populations [24-28]. Further, for self-measured values to be clinically useful, they need to be reported accurately. Several studies in different study populations have shown that caution is warranted when using patient-reported data for making clinical decisions as a considerable number of patients report values that do not sufficiently represent their actual measurements [29-34].

To the best of our knowledge, no studies have assessed the reliability and accuracy of patient-generated creatinine data or looked at the level of adherence to a protocol of self-monitoring creatinine. This is unfortunate, as the introduction of self-monitoring offers a good opportunity to improve post-transplantation care. Our first research goal was to investigate the level of adherence of kidney transplant patients to a creatinine monitoring schedule. Our second research goal was to determine the reliability of the creatinine values that were registered in a Web-based self-management support system (SMSS). As this SMSS automatically provided instructions for further actions (eg, continue regular schedule or contact the hospital) upon registration of a new creatinine value, our final research goal was to determine whether patients adhered to the system’s instructions.

## Methods

### Patients and Study Design

The data used in this study were obtained from the ADMIRE project (Assessment of a Disease management system with Medical devices In REnal disease), a cooperation between the Leiden University Medical Centre (LUMC), the Technical University of Delft, and the Dutch Organization for Applied Scientific Research (registered in the Dutch Trial Register: NTR3548). This extensive project comprised the technical development of an SMSS in which several studies were performed to optimize the system to suit patients’ needs and wishes, as well as a prospective randomized controlled trial (RCT) to study whether self-monitoring kidney function supported by an SMSS can replace part of regular outpatient care without compromising on the quality of care. The study protocol was approved by the Medical Ethics Committee of the LUMC.

Patients were eligible for participation in the RCT if they were about to receive a donor kidney or recently received one, were ≥18 years of age, mastered the Dutch language sufficiently, had access to the Internet, and had a creatinine level of ≤300 µmol/l within 4 weeks post transplantation. Patients were excluded if they were visually impaired or were considered ineligible by their treating physician (eg, due to mental retardation, a history of noncompliance to treatment). We therefore had a selection of patients that seemed most capable for engaging in self-monitoring.

Recruitment of living donor recipients took place during a pretransplant consultation with a nurse-practitioner. Recipients of a postmortem kidney were recruited during their post-transplantation stay in the hospital by the primary investigator (CvL). After this face-to-face introduction, patients received a written explanation of the study with an informed consent form. If a signed informed consent was not returned within 2 weeks from the recruitment date, patients were contacted telephonically to inquire whether they were interested in participating. After signing informed consent, each participant was assigned a study number. Incoming informed consents were treated in consecutive order. Study numbers were allocated to either the intervention or the control group according to a preset randomization schedule which was created by a medical statistician. The randomization procedure was blinded for the project members directly involved in patient recruitment.

For this study, only participants randomized to the intervention group were included.

### Intervention

#### Devices and Self-Management Support System

For self-monitoring creatinine, each participant received a StatSensor Xpress-i Creatinine Meter (Nova Biomedical, Waltham, USA) and related test material (ie, test strips, control solution to test the quality of the strips, and safety lancets for capillary blood sampling). On the basis of a drop of blood of 1.2 μL, the StatSensor can show either current level of creatinine or estimated glomerular filtration rate (eGFR). At our medical center, clinicians usually communicate level of creatinine to kidney transplant patients, and the device was set to show creatinine.

For self-monitoring blood pressure, each participant received a Microlife WatchBP Home (Microlife, Heerbrugg, Switzerland), an oscillometric device for blood pressure self-measurement on the upper arm. Both devices had a memory function and the option to download stored values to a computer.

**Figure 1:**
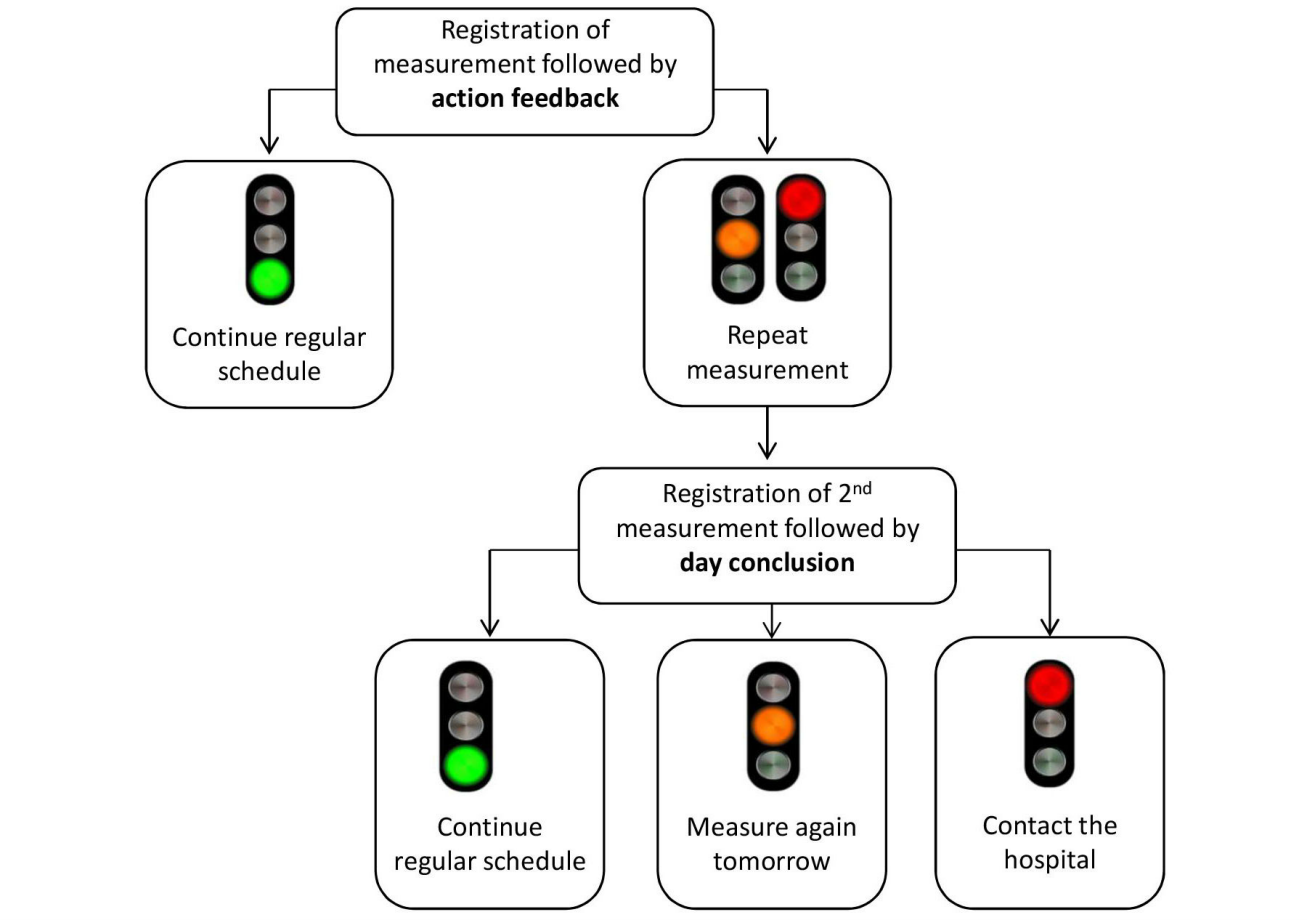
Possible feedback combinations in the Self-Management Support System (SMSS).

A Web-based SMSS was available for all patients in the intervention group. This SMSS entailed an e-learning module instructing patients on how to use the SMSS system, that is (a) how to perform creatinine measurements at home, (b) how to register self-measured values in the SMSS (both creatinine and blood pressure), and (c) how to respond to messages from the automatic feedback system to support patients’ interpretation of the creatinine trends. Figure 1 provides an overview of the feedback process. The feedback appeared directly after registration of a new creatinine value and consisted of a traffic light with corresponding text. Per day, a maximum of two creatinine values could be registered. After registration of the first value of the day, a green light indicated that there was no reason for concern and was associated with the advice to just continue regular measurement frequency. This was termed the day conclusion, as no further actions were required for the concerning day. The appearance of an orange or red light (in case the newly registered value was respectively >15% or >20% higher than mean of the previous five values) directly after registration of the first value of the day indicated that there was some reason for concern. The system’s advice was then to perform and register a second measurement to confirm the first measurement. This was termed action feedback, as it required an immediate action. After registration of a second measurement, an appearing green light indicated that there was no further reason for concern. In this case, patients were advised to continue their regular monitoring frequency. Alternatively, an orange light indicated that there was some reason for concern and patients were advised to measure again tomorrow. Finally, a red light indicated that there was reason for concern and patients were advised to contact the hospital. Feedback given after the registration of a second measurement was also termed day conclusion, as no further values could be registered. See Figure 1 for an overview of all possible feedback combinations.

A link was created between the SMSS and the electronic hospital system in order for the registered creatinine values to be visible for the treating nephrologist(s). Nephrologists did not receive a copy of the supplied SMSS feedback.

#### Procedure

The time schedule for providing instructions depended on whether patients participated in the living donor program or whether they received a kidney from a deceased donor. Two weeks before a scheduled transplantation, patients received account details to log in to the SMSS and use the e-learning module to prepare for self-monitoring. Recipients of a kidney from a deceased donor received account details during their post-transplantation stay in the hospital. A laptop was available to use the e-learning modules. After being virtually instructed through e-learning, all patients received the creatinine device and supplementary face-to-face instructions. Special attention was paid to the fact that patients had to take action themselves upon the system’s feedback, as their nephrologist(s) would only check the home-based creatinine values in advance of or during an outpatient visit or a telephonic consult. Patients were then encouraged to practice using the creatinine device during the remainder of their hospital stay.

Home-based creatinine measurements had to be performed according to a fixed frequency, being daily during the first 4 weeks (phase 1), every other day for weeks 5-9 (phase 2), twice a week for weeks 10-15 (phase 3), and weekly from week 16 onward (phase 4). This scheme was based upon the usual frequency of laboratory testing, which decreases when time since transplantation increases. However, as the creatinine device tends to be less accurate than laboratory tests [35], the usual frequency of laboratory testing was multiplied with a factor 7 to obtain a more reliable trend. After measuring, patients registered the results in the SMSS.

In addition to conducting regular creatinine measurements, patients were advised to perform a test measurement when opening a new bottle of test strips. These measurements could be termed test measurements by pressing a designated button on the creatinine device.

From week 8 after transplantation on, every other face-to-face outpatient visit with regular hospital-based laboratory measurements was replaced by a telephonic consult to discuss self-monitored creatinine and blood pressure. Although regular face-to-face visits also include other laboratory measurements (eg, trough levels of immunosuppressive medication), these analyses do not need to be performed in the same frequency as for creatinine due to their (expected) little variation over a short period of time. It was therefore deemed unnecessary to replace these other laboratory measurements with a home-based alternative.

To remind nephrologists of scheduling a telephone consult instead of a face-to-face visit, a short note asking for the next appointment to be a telephonic one was shown repetitively in a patient’s electronic hospital file. It was, however, up to the treating nephrologist to judge whether a patient’s condition allowed for a telephonic consult to take place or whether a face-to-face visit was needed.

At the end of the intervention period of one year, all patients were invited to bring their creatinine device to download logged data. This data included test results, date and time of all performed measurements, and, if applicable, an indication of whether a specific value was termed a test measurement. Further, data that were automatically logged in the SMSS were downloaded including the registered value(s), date of performed measurement (according to the patient), date of registration, and the feedback that was supplied after each newly registered creatinine value.

### Measures

Patients completed a questionnaire at baseline to collect demographic characteristics. The read-out data from the creatinine device and the data that were logged in the SMSS were combined using date of measurement. For the creatinine device, measurement date was the date of measurement performance that was registered automatically in the device memory. For the SMSS, measurement date was the date of measurement performance according to the patient.

### Statistical Analyses

#### Adherence to Measurement Frequency

To assess whether patients adhered to the measurement protocol, we separated adherence according to device-logged data (did patients perform the requested number of measurements?) and adherence to SMSS-logged data (did patients register the requested number of measurements in the SMSS?). If applicable, paired *t* tests were conducted to compare means using SPSS 22.0 for Windows (IBM Corp, Armonk, NY). In these cases *P*<.05 was considered statistically significant. For adherence according to device-logged data, we calculated the number of days with measurements per patient per phase and compared this with the number of requested measurement days. Number of requested measurement days was 28 during phase 1 (4 weeks), 15 during phase 2 (5 weeks), 12 during phase 3 (6 weeks), and 37 during phase 4 (37 weeks). To make it easier to interpret the results, level of adherence was divided in four subcategories for this study:

1. Extremely nonadherent: measurements performed during less than 25% of the requested days
2. Nonadherent: measurements performed during 25-74% of the requested measurement days
3. Adherent: measurements performed during 75-100% of the requested measurement days
4. Overadherent: measurements performed more frequently than requested (ie, > 100%).

This same procedure was used to calculate the level of adherence to registration of measurement in the SMSS, that is, whether patients registered measurements on the requested number of days.

#### Moment of Registration

Date of measurement (derived from device-logged data) was compared with the date of registration of this measurement (derived from SMSS-logged data). Per patient the average number of days delay between measurement and registration was calculated. Furthermore, we investigated whether delayed registration was related to the stability of creatinine level by comparing feedback that was generated by the SMSS in case of registration on day of measurement with feedback that was generated when registration was delayed.

#### Reliability: Correctness and Representativeness of Registered Data

The reliability of registered data is determined by both the correctness and the representativeness of registered values. To study correctness of the registered data, we investigated the one-on-one correspondence between registered and measured values on a given day. Three different categories were distinguished:

1. Reliable SMSS registrations, in case a value that was registered in the SMSS corresponded to the device logged value on a given date. Only days with an equal number of measurements logged in the device and SMSS were taken into account.
2. Noncorrespondence, in case an SMSS registered value did *not* correspond to the device-logged value on a given date. Only days with an equal number of measurements in the device-logged and SMSS-logged data were selected. All cases of noncorrespondence were carefully checked for potential causes of the deviance (eg, wrong combination of date and measured value, typo, rounding off). The cases where no potential cause was found were termed incorrect entries. For each patient, a mean level of creatinine was calculated for the values that were actually measured and for the values that were registered using cases of incorrect entry only. A paired *t* test was performed to compare these means. Total and median number of noncorresponding values was calculated per patient. Patients with a high number of noncorresponding values were selected for further exploration.
3. Phantom values, in case a value was registered in the SMSS on a given date while according to the data stored in the device no measurement was performed on that specific date. All potential phantom values were thoroughly checked for alternative explanations before it was concluded that there was no relation with values that had been measured by the patient. A paired *t* test was performed using the mean of the phantom values versus the mean of all measured creatinine values per patient.

Furthermore, to get a reliable impression of a creatinine level over time (trend), the SMSS registered values need to represent what was actually measured. It is therefore important to know how often a measured value was not registered in the SMSS and whether the unregistered values differed in any way from the registered values. The measured values not being registered in the SMSS were split into two categories:

1. Omissions, in case one or more measurements were performed on a given date, but no value was registered in the SMSS. Total and median number of omissions per patient was calculated. For each patient, we calculated a mean level of creatinine for the values that were both measured and registered and a mean level of creatinine for the values that were measured on days without any registration. A paired *t* test was performed to compare these means.
2. Selection of measurements, which is the case when the number of performed measurements that is stored in the device is higher than the number of registered measurements on a given date. Therefore only days with a difference between the number of measured and the number of registered creatinine values were selected (eg, three measurements stored in the device and one value registered in the SMSS). We then calculated per patient the mean of all values stored in the device *and* registered in the SMSS and the mean of all values stored in the device, *but not* registered in the SMSS. A paired *t* test was performed to compare these means.

#### Adherence to Feedback

After registration of a creatinine value in the SMSS, patients received an automatically generated advice on the necessary action to take (see Figure 1). To investigate the level of adherence to the advice generated by the SMSS, we separated between adherence to action feedback (supplied after the registration of a first measurement when further action was required) and adherence to the day conclusion (supplied when no further actions were required after the first registration of a day or when a second and final measurement was registered on the same day).

Action feedback could only appear in case the newly registered creatinine value was higher than the previous ones and required an additional measurement to confirm the first. In these cases, the feedback system of the SMSS showed an orange or red traffic light with the corresponding advice to repeat the measurement. From the SMSS-logged data, we selected those cases where a second measurement was requested and checked whether the concerning patients indeed measured and registered a second creatinine value on the same day.

To study adherence to the day conclusion, we only considered the cases in which patients again were confronted with an orange or a red traffic light. In case of a request to perform another measurement the next day (orange traffic light), the SMSS-logged data were checked to see whether the requested action was indeed performed. In case of a request to contact the hospital (red traffic light), patient hospital records were searched for telephonic and outpatient contacts on dates following the concerning feedback.

### Sensitivity Analyses

Two sensitivity analyses were performed to control for potential bias. First, as being hospitalized limits the possibility to keep up with requested measurement and registration frequency, the level of adherence was analyzed with and without patients that were hospitalized during the study. The second sensitivity analysis concerned the test measurements patients were requested to perform when opening a new bottle of strips. Many patients either did not perform test measurements or did not indicate them as such. To prevent test values to be mistakenly considered creatinine measurements, all values that were stored in the device memory were checked. Potential test values were discussed and decided upon by the two main authors. The following criteria were used: (1) the value was not registered in the SMSS, (2) the value differed from the previous and following value, (3) the value fell within the test value range that was set by the manufacturer (133-239 µmol/l), and (4) the value followed or was followed by at least one SMSS-registered value measured on that same day (measured shortly after one another according to device-logged data). After having thoroughly checked and discussed all potential test values, for 24 values it remained unclear whether they were test values or not. We therefore performed all analyses concerning the representativeness of registered creatinine data with and without these 24 values.

Furthermore, we compared our findings concerning patient self-monitoring creatinine to a more broadly used and well-accepted form of patient monitoring, being self-monitoring of blood pressure. For this purpose, we performed two analyses with the self-monitored and self-reported blood pressure measurements in our study population. First, we looked at adherence to the blood pressure measurement protocol using the same procedure as for creatinine: number of days with measurements versus number of requested measurement days per patient per phase. The requested frequency of blood pressure measurements was equal to the measurement frequency of creatinine. As many patients used other blood pressure devices than the device we supplied for the study, we could not determine adherence to the measurement protocol in a reliable way. We therefore only could assess adherence to the registration protocol by comparing the number of registered blood pressure measurements (SMSS-logged data) with the requested measurement frequency.

Second, we looked at correspondence between measured and registered blood pressures. To prevent potential bias and misinterpretation, we (1) only used measurements from days with an equal number of measurements and registrations and (2) calculated a mean arterial pressure (MAP, [(2×diastolic)+systolic]/3) for both measured and registered blood pressures per day. Using all cases of noncorrespondence between measured and registered MAP, an overall MAP was calculated per patient for both blood pressures that were actually measured and for blood pressures that were registered in the SMSS. A paired *t* test was performed comparing these means.

## Results

### Participants

Within the period of inclusion, in total 217 patients received a kidney transplant of which 155 were considered eligible for participation. The main reasons for ineligibility were insufficient mastery of the Dutch language (25/62, 40%) and no access to a computer or limited computer skills (16/62, 26%). In total, 119 patients (76.7%) signed an informed consent. The main reason for not wanting to participate was the anticipated burden of self-monitoring (17/36, 47%). A total of 65 patients were randomized to the intervention group. After randomization, 3 patients dropped out because of graft dysfunction, death, and cancellation of transplantation (none was study related). Before starting to self-monitor kidney function at home, 4 patients canceled their participation because they reported having little trust in the creatinine device, experienced difficulties when logging into the SMSS, experienced business rush, or had a worsened condition post transplantation. In total, 58 patients were supplied with a creatinine and blood pressure device of which 4 never performed any measurement.

**Table 1:** Baseline patient characteristics.

Characteristics^a^		Received devices (n=58)	Used devices and SMSS during all phases (n=43)		*P*
Sex, male, n (%)		37 (64)	27 (63)		*P*>.99
Age at transplantation, mean (SD)		51.6 (14)	52.5 (15)		*P*=.40
Living together or married, n (%)		43 (74)	33 (77)		*P*=.50
Children, yes, n (%)		39 (67)	29 (67)		*P*=.76
**Educational level, n (%)**					*P*=.25
	Low	22 (38)	14 (33)		
	Middle	16 (28)	12 (28)		
	High	20 (34)	17 (39)		
Paid job, yes, n (%)		31 (53)	23 (53)		*P*>.99
Origin, native, n (%)		53 (91)	41 (95)		*P*=.10
Former transplantation, n (%)		6 (10.3)	4 (9.3)		*P*=.64
Dialysis dependence before transplantation, n (%)		26 (44.8)	21 (49)		*P*=.14
Living transplantation, n (%)		50 (86.2)	38 (89)		*P*=.52
Kidney function (eGFR), mean (SD)		49 (16.1)	50 (15)		*P*=.34

^a^For a few patients, data on marital status and education were missing. These data were imputed in SPSS using multiple imputation (10 imputations).

**Figure 2:**
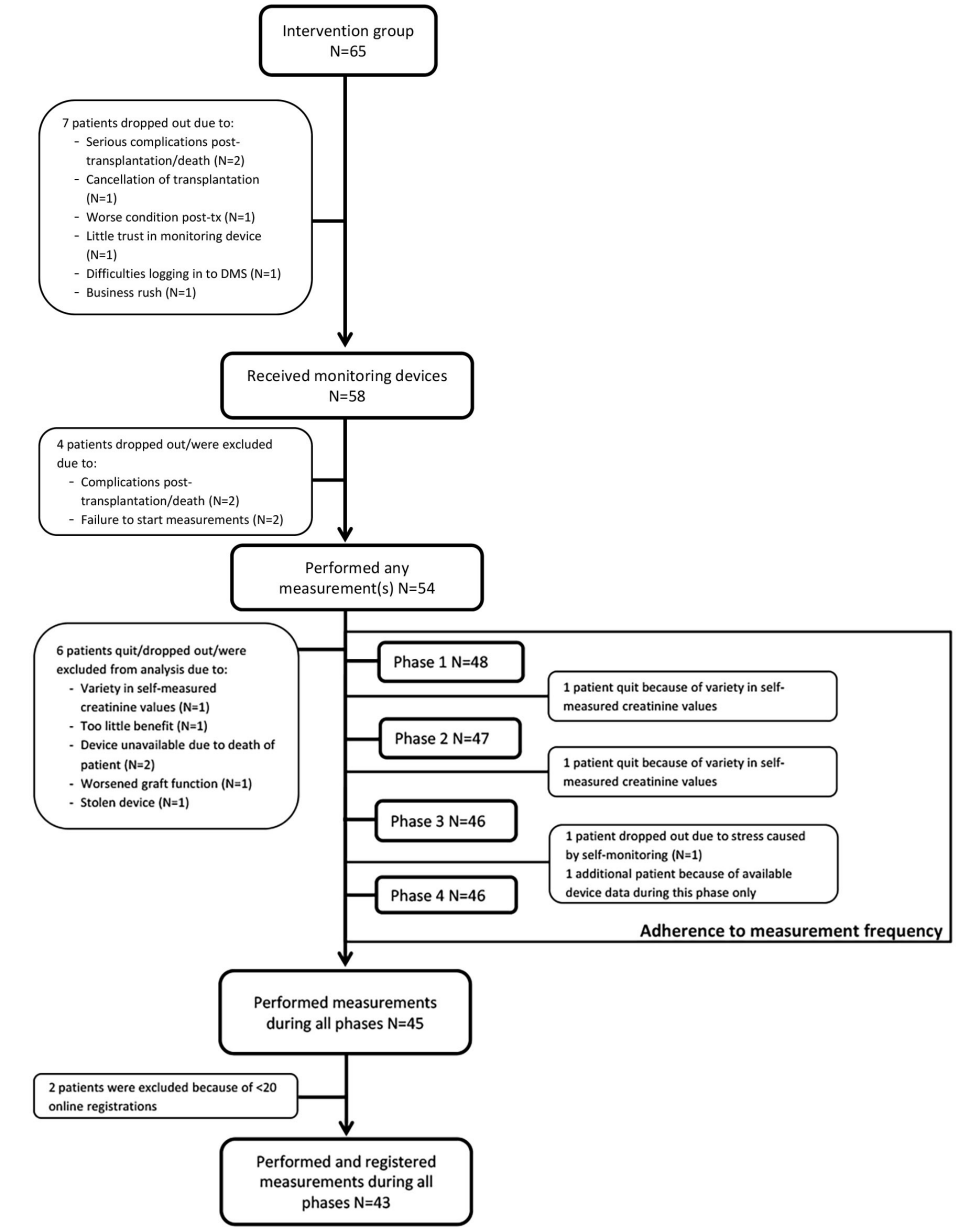
Study flowchart.

To study the level of adherence to requested measurement frequency, we included patients of whom measured values were available for at least one complete study phase (N=48). To study the reliability of registered data and adherence to system feedback, we included patients who performed and registered measurements during all study phases (N=43). The flowchart in Figure 2 gives a stepwise overview of the patient flow and for which selection of patients a specific analysis was performed.

Patient characteristics are shown in Table 1. The mean age of participants was 52 and 53 years for patients who received the monitoring devices (N=58) and patients who performed and registered measurements during all study phases (N=43), respectively. The number of patients with both a low and a high educational level was slightly higher than in the average Dutch population [36]. Almost 90% of our participants (50/58) had received a kidney from a living donor, while the ratio of transplantations with living versus postmortem kidneys was about equal in our center during the period of inclusion. This discrepancy is mainly due to a higher percentage of ineligibility among recipients of a postmortem versus living kidney: 51% versus 16%, respectively.

**Figure 3:**
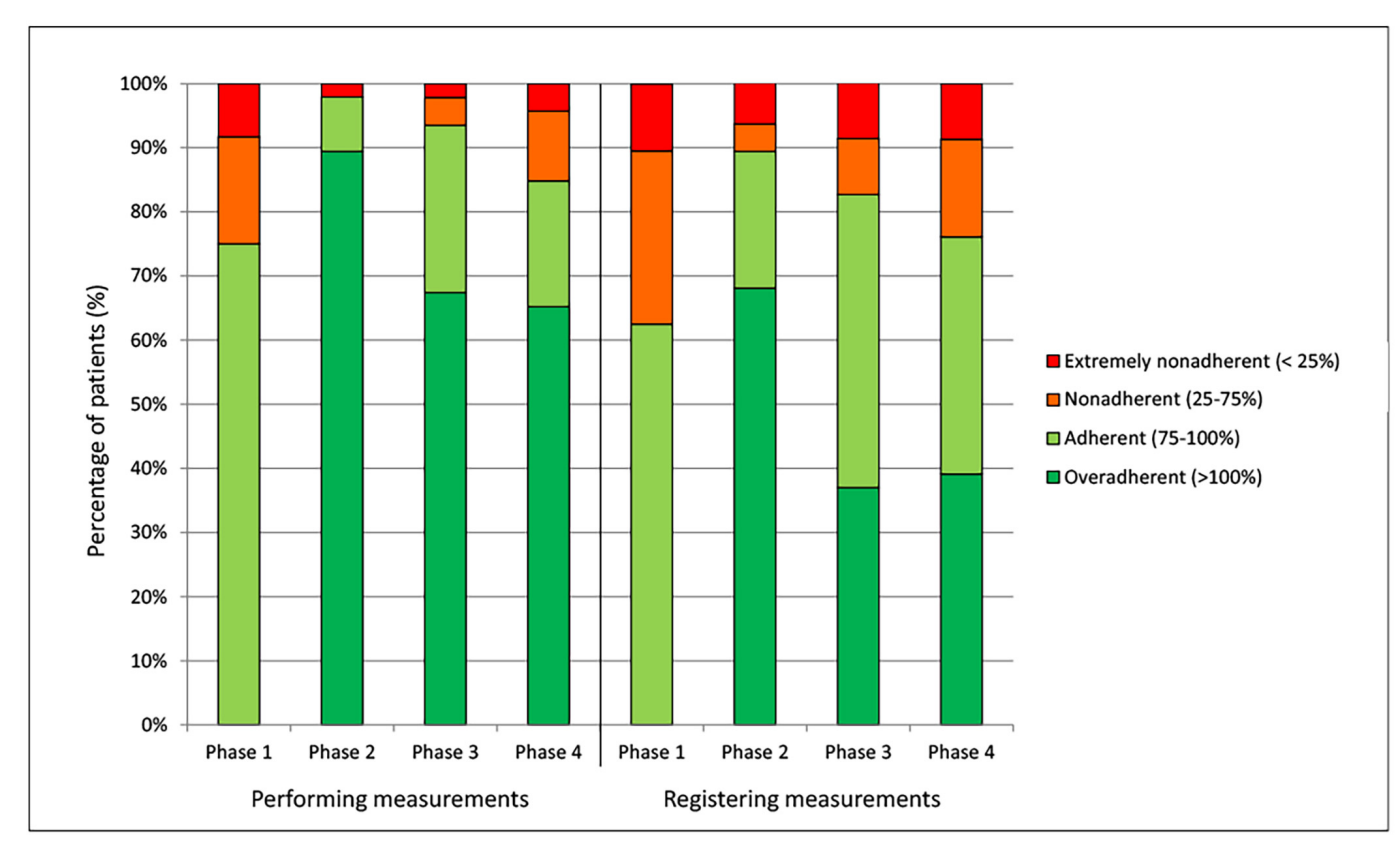
Patient adherence to requested measurement and Self-Management Support System (SMSS) registration frequency per study phase. The x-axis refers to the different measurement frequencies requested throughout the study for both performed and registered measurements (daily, every other day, twice a week, and weekly in phases 1, 2, 3, and 4, respectively), and the y-axis shows the percentage of adherent and overadherent and nonadherent and extremely nonadherent patients.

**Figure 4:**
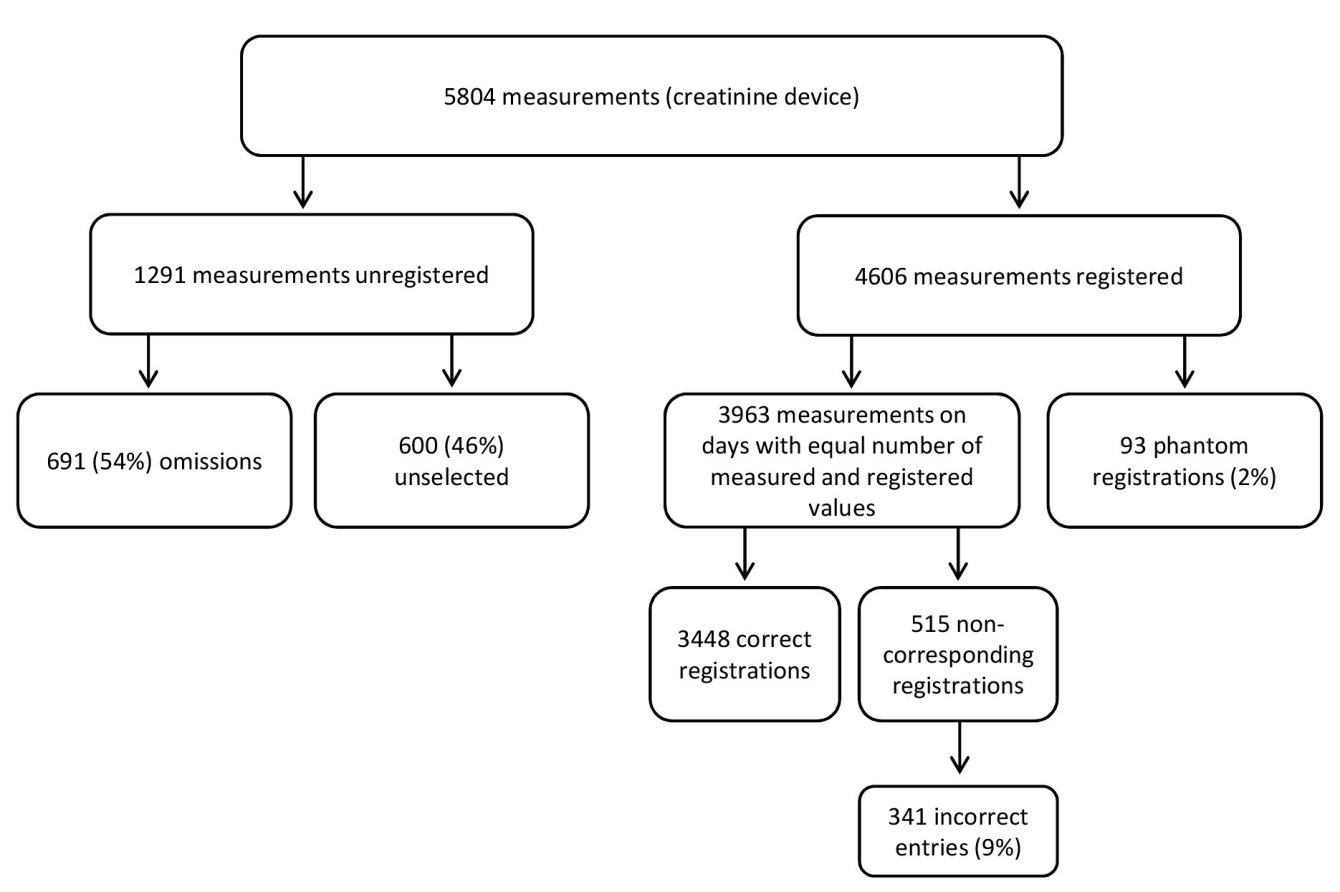
Reliability of creatinine values registered in the disease management system. The unregistered (1291) and registered values (4606) do not add up to the total number of measurements in the device (5804) due to the presence of phantom values.

No differences were found between patients who received the monitoring devices and patients who performed and registered measurements during all study phases for the characteristics we measured at baseline (see Table 1).

### Adherence to Requested Measurement Frequency

Adherence to requested measurement frequency according to device-logged data (did patients perform the requested number of measurements?) and adherence to SMSS-logged data (if patients performed a measured, did they register the requested number of measurements?) are shown in Figure 3. During phase 1 (daily measurements), adherence to the requested number of creatinine measurements was lowest with 75% (36/48). Subsequently, adherence rose to over 90% during phases 2 (46/47, measuring every other day) and 3 (43/46, measuring twice a week), and then decreased to 85% during phase 4 (39/46, measuring weekly). In total, 4 patients performed less than 75% of the requested measurements throughout two or more phases. For registration of the requested number of creatinine measurements this same pattern is shown, although the percentage of nonadherent patients is higher during all phases. In total, 8 patients were nonadherent during two or more phases regarding registration of the requested number of measurements.

To control for the potential influence of hospitalization on the level of adherence to measurement and registration protocol, we repeated our analysis with all hospitalized patients excluded. The total number of hospitalized patients was 11 during phase 1, 3 during phase 2, 7 during phase 3, and 9 during phase 4. Excluding these patients did not change our initial findings.

### Moment of Registration

When looking at the date of measurement versus the date of registration of measurements, a mean delay of 4 days (SD 10) was found. The level of delay varied from 1 to 81 days. A total of 7 patients (7/47, 15%) always registered their test results on the day of measurement and 15 patients (31%) had an overall mean delay of less than 1 day between measurement and registration. In total, 22 patients (46%) had an overall mean delay of more than 3 days, ranging up to a mean difference of 29 days between the date of measurement and the date of registration. One could hypothesize that patients do not feel the need to register their measurement if their level of creatinine is stable. However, the feedback that was generated by the SMSS for measurements registered on the day of measurement versus measurements that were registered with delay did not differ: in both situations, patients were requested to repeat the measurement in about 7% of all registrations.

The x-axis refers to the different measurement frequencies requested throughout the study for both performed and registered measurements (daily, every other day, twice a week, and weekly in phases 1, 2, 3, and 4, respectively), and the y-axis shows the percentage of adherent and overadherent and nonadherent and extremely nonadherent patients.

### Reliability of Registered Data

Of the 43 patients included in the reliability analysis, the total number of values stored in the creatinine devices was 5779 and the total number of values registered in the SMSS was 4606. To investigate correspondence between measurement and registration, only days with an equal number of measurements in the device and registrations in the SMSS were selected. Total number of measurements performed and registered on these days was 3963. Figure 4 gives an overview of the reliability of the registered data, showing that 3448 (87.00%) of these values were registered correctly.

#### Noncorresponding Registrations

In 13% (515/3963) of all creatinine registrations, the registered value did not correspond to the value that was measured on that day. In 174 cases, we could determine the origin of the difference (eg, wrong combination of date and measured value, typo, rounding off). The remaining 341 registrations (9% of all registered values) were used for further analysis. Median number of noncorresponding values per patient was 3 (Interquartile range, IQR 8). Overall, 11 out of 43 patients (25%) made no mistakes at all, while another 11 patients made more than 10 mistakes. In total, 2 patients had an extremely high number of noncorresponding registrations. The first one had 52 noncorresponding registrations, half of which were found to be exactly 10, 20, 30, or 40 µmol/l lower than what was actually measured. In total, 83% (43/52) of his noncorresponding entries were lower than what was measured. The other patient registered 92 noncorresponding values, which were lower than the actual measured values in 93% (86/92) of his cases.

In case of noncorrespondence, the difference between measured and registered ranged from 1 to 73 mmol/l with a median of 9 mmol/l (IQR 13). The noncorresponding registrations were significantly lower than the actual measured ones: 123 mmol/l (SD 28) versus 130 mmol (SD 33), respectively (*t*_340_=8.7, *P*≤.001).

#### Phantom Values

In total, 93 phantom values were found, which was 2.02% of all registered values (N=4606). In total, 30% of patient (13/43) registered at least one phantom value, 14% (6/43) registered seven or more. Of the phantom values, 20 resembled the measurements of surrounding days, which would suggest these phantom values were only registered to adhere to the registration protocol. However, this appeared not to be the case as 16 of these 20 resembling phantom values were registered by a single patient who would already have been overly adherent without these phantom values. Overall, 3 patients registered creatinine values during the months where no measurements were logged in the device. For example, one patient quit measuring in February, but registered three measurements during March and April. Phantom values were significantly lower than actually measured ones, respectively 107 (SD 26) and 123 (15) mmol/l (*t*_11_=3.9, *P=*.003).

#### Representation of Registered Creatinine Values

Of the 5779 measurements found in the creatinine device-logged data, 1300 values (22.49%) were not registered in the SMSS. In 700 cases, one or more measurements were performed on a given date, but no value was registered in the SMSS (ie, omission). Number of omissions per patient ranged from 0 to 145; 5 patients had no omissions at all and 11 patients had omitted 20 values or more. Median number of omissions was 8. The omitted values were significantly higher than the registered ones (mean of 139 [SD 31] vs 130 [SD 32] µmol/l, respectively (*t*_42_=−3.8, *P* ≤.001).

In several cases, more measurements were performed per day than values were registered. In these so-called measurement series, the number of performed measurements ranged from 2 to 8 with a median of 2 (IQR 1) per day. The total number of values that was measured within a series of measurements but was not registered in the SMSS was 600. The mean of the creatinine values that were both stored in the device and registered in the SMSS was significantly lower than the mean of the creatinine values that were stored in the device but not registered in the SMSS (unselected for registration): 137 (SD 35) vs 143 µmol/l (SD 36), respectively (*t*_42_=−2.5, *P*=.02).

Repeating both analyses with the 24 values included that could either be test values or regular measurements did not change our findings (data not shown).

### Adherence to System Feedback

**Figure 5:**
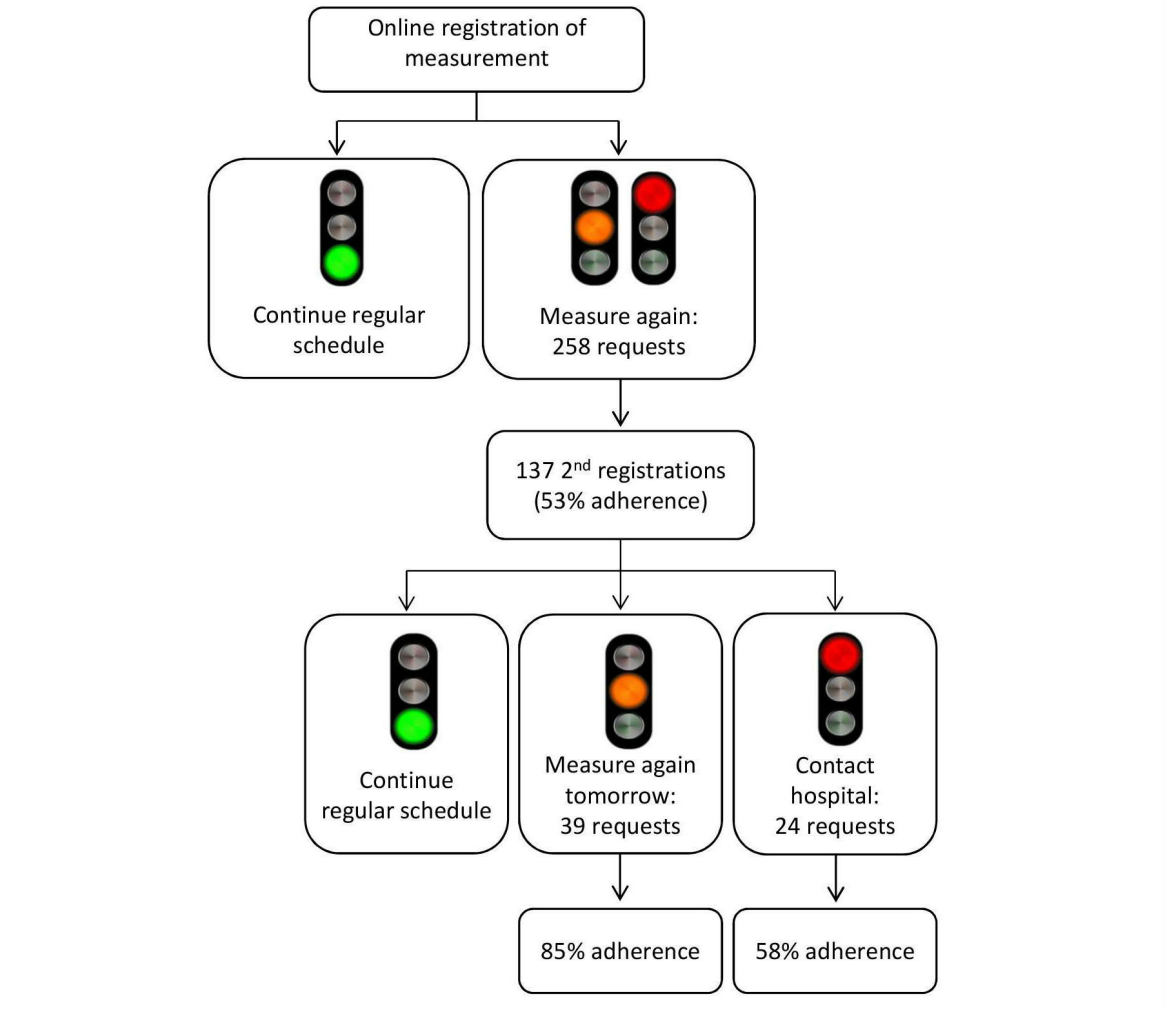
Patient adherence to system feedback.

Results are given separately for adherence to the action code (requesting patients to perform a second measurement directly) and adherence to the day conclusion (feedback that only appeared in case a second measurement was requested and registered). An overview of the feedback procedure and level of adherence to the different kinds of feedback is shown in Figure 5.

#### Adherence to Action Code

In 258 cases, patients were requested to perform a second measurement directly. In 137 cases (53%), patients actually performed and registered a second measurement. In 85 cases (33%), date of registration differed from the date of measurement, suggesting that delayed registration was the main reason for not adhering to feedback, as this feedback was no longer up-to-date when shown to the patient. In 14 cases (5%), multiple measurements were performed in advance of registration. As these multiple measurements were probably representative of actual creatinine level already, patients might not have felt the need to perform another one.

#### Adherence to Day Conclusion

In 39 cases, patients were requested to measure again the next day. In 33 cases (85%), this feedback was adhered to. In 3 cases (8%), the measurements were registered several days after measurement (delayed registration), suggesting that the feedback to measure again the next day was no longer up-to-date when shown to these patients. In one case, the requested measurement was performed, but was not registered in the SMSS.

The advice to contact the hospital was given 24 times, which was followed up 14 times (58%). In the remaining 10 cases (concerning 10 individual patients), measurements were registered with several days’ delay. As feedback was no longer up-to-date when shown to the concerning patients, this suggests delayed registration was the main reason for not adhering to the feedback to contact the hospital. If only cases with registration on the day of measurement were taken into account, adherence to contacting the hospital was 100%.

### Sensitivity Analysis: Adherence to and Reliability of Blood Pressure Measurements

In total, 31 blood pressure devices could be read out. The total number of values found in the 31 available pressure devices was 4917, and the total number of values registered in the SMSS was 5637. The higher number of registered than measured blood pressures is due to patients using multiple blood pressure devices during study participation, while data of only one device were available. Adherence to registration of blood pressure measurements was comparable to creatinine registrations with 70% (34/48), 88% (41/47), 87% (40/46), and 81% (37/46) of patients registering >75% of the requested number of measurements during phases 1, 2, 3, and 4, respectively. Although the percentage of registrations not corresponding to the measured MAP was comparable to what we found for creatinine (14% vs 13%, respectively), we could not replicate the significant difference between registered and actually measured creatinine for blood pressure: 97 mmHg (SD 2) versus 96 mmHg (SD 9) for registered and measured MAPs, respectively (*t*_3_=.20, *P*=.84).

## Discussion

### Principal Findings

Self-monitoring kidney function and blood pressure at home offers important advantages for patients after kidney transplantation. However, the value and safety of self-monitoring depend on how well patients actually adhere to their self-monitoring tasks, the reliability of the test results they report, and whether they take appropriate actions based on their measurements. This study showed that the level of adherence was generally good. Well above 90% of all patients performed the requested number of measurements during months 2-4 post transplantation. Adherence was lower during the first month when more measurements were requested and during months 5-12 post transplantation when less measurements were requested, with about 75% and 85% of patients adhering to the requested number of measurements, respectively. Overall adherence to registration of measurements was about 10% lower than adherence to performance of measurements during all phases. Two studies reporting on the level of adherence to monitoring vital signs after lung transplantation found similar percentages of adherence being above 80% for the entire study period [24,26]. For self-monitoring blood pressure, patients with uncontrolled hypertension were shown to be adherent for about 73% of the entire study period [25,29]. In both studies, level of adherence was highest in the first few weeks and declined gradually over time. In sum, mean level of adherence that has been found in this study corresponds to percentages that have previously been reported. In contrast, we did not find the highest levels of adherence in the first period. This may have been due to a strenuous measurement protocol. Patients had to measure every day in the first month. In these first weeks when patients have to recover and have to get used to life post transplantation, performing measurements in such a high frequency might be too burdensome. Furthermore, in this first period, face-to-face visits were not yet replaced by telephonic consults and patients therefore visited the hospital at least weekly to monitor early signs of graft failure. Due to this high frequency of visits, patients may have felt a reduced need to perform measurements at home, as they did not have to rely on these measurements. The latter may also be an explanation for nonadherence during the whole study period.

Furthermore, for self-monitoring to be a safe alternative to regular face-to-face follow up, patient-reported test results need to be accurate. In this study, approximately 90% of both creatinine and blood pressure measurements were registered correctly in the SMSS. This percentage corresponds to what has previously been described for patient-reported blood pressure [29,37] and anticoagulation [34] and is much higher than has been observed for patient-reported levels of blood glucose. A study by Kalergis and colleagues [30], for example, showed that slightly over half of the total group of patients with either diabetes type 1 or 2 was considered very reliable in their reporting. For patients with diabetes type 2 and for pregnant women self-monitoring blood glucose, some studies even showed that the majority of patient-reported data was unreliable [31,32].

In cases of noncorrespondence between measured and actually registered values, values that were eventually registered in the SMSS were significantly lower than those actually measured. These results seem to suggest that patients select, alter, or add values in such a way that their creatinine profile looks more positive. This corresponds to what has been found in a population of patients with thrombosis, where the percentage of time when patients’ level of anticoagulation was within the desired range was significantly higher when using patient-reported data compared with data stored in the device [34]. For patients with diabetes or hypertension, it was found that inaccurate reporting increased with increasing levels of blood glucose [31] or blood pressure [37]. Why patients report values that look better than the actual measured values or add nonexistent measurements has not yet been fully clarified. For diabetes, it has been suggested that patients report false glucose levels due to a feeling of guilt for not having achieved glycemic goals [32] or add phantoms values in an attempt to fill up logbooks and satisfy their health care providers [30]. Both situations seem to represent an attempt to be a “good” patient. However, altering and selecting data that are not representative of the actual clinical situation or adding phantom values in any case may be dangerous. This can lead to suboptimal treatment and, eventually, to worsened patient outcomes [30,37]. In a study by Kendrick and colleagues, it was found indeed that women with pregnancy-derived diabetes received suboptimal treatment due to a large difference between their reported glucose values and what they had measured [32]. Results of another study showed that diabetic patients who were more reliable in their reporting had a significantly better glycemic control. It was suggested that this may be due to the ability of clinicians to adjust therapy more precisely if measurements are reported accurately [30]. To prevent incorrect reporting, it has been recommended to rely on the memory capacity of measurement devices, preferably by using devices that can transfer data automatically [30,31,33,34].

Besides eliminating the occurrence of both intentional and unintentional errors, the automatic transfer of data offers a solution for the observation that patients seem to save up their measurements before registering them. Many patients saved up their measurements over several days or even weeks to register them all at once. More than one-third of our participants displayed a mean delay of 5 or more days between measurement and registration of data. This is alarming as frequent monitoring and taking immediate action in case of early signs of graft failure is vital to prevent or diminish damage to the kidney transplant. An explanation for saving up measurements before registering them might be that the measured creatinine values remained stable. However, patients seemed to postpone registration regardless of the stability of their kidney function. Indeed, postponement of registration appeared to be the main reason why patients had not followed up the advice to contact the hospital when creatinine levels had alarmingly increased by over 15%. Patients’ perception of these significant increases could have been influenced by the fact that the innovative device that was used during this study tended to be less accurate than hospital laboratory measurements [35]. As a consequence, patients might have been inclined to attribute sudden increases in level of creatinine to a technical imprecision of the device.

### Strengths and Limitations

To the best of our knowledge, this is the first study to assess adherence to a protocol of self-monitoring creatinine and to investigate the accuracy and reliability of patient-generated creatinine data. Enabling patients to self-monitor kidney function at home would have important advantages, especially for patients living in remote areas. There are, however, some limitations that must be considered when interpreting our findings. First, the study was conducted at a single institute. As each hospital has its own way of delivering care, results might be different when conducted in other institutions. Our findings do, however, resemble what has previously been found in other disease populations. Second, participation in this study was voluntary and we selected patients with access to the Internet. Therefore, it is possible that our patients had an above-average motivation to self-monitor. It is therefore likely that the current findings provide a conservative estimate of the true incidence of nonadherence and inaccuracy. In line with this, patients reported very strong intentions to engage in self-monitoring both at the start and after 4 months into the trial [38]. Their intention was found to be especially associated with their overall affective reaction toward using the system [38]. A considerable number of eligible patients had to be contacted to inform whether they were interested in study participation instead of giving informed consent immediately. However, the limited variance found in the level of intention to engage in self-monitoring suggests that patients who had to be contacted were not more hesitant to engage in self-monitoring than patients who provided immediate informed consent.

Finally, the way adherence and nonadherence were determined is arbitrary to some extent. As the importance of frequent and very frequent monitoring differs per subpopulation and parameter of interest, no gold standard for what can be considered adherent is available. High blood pressure, for example, needs to be present over a longer period of time before becoming detrimental, while an increasing level of creatinine can be indicative of a rejection episode, leading to irreversible damage or even loss of the transplanted kidney if not quickly noticed.

### Implications

This study shows that the level of adherence to a protocol of self-monitoring creatinine in the first year after kidney transplantation was generally good, although adherence declined over time. In addition, our results suggest that measuring every day in the first period after transplantation might be too burdensome. Furthermore, 90% of data were shown to be accurately reported. In line with previous findings, however, several patients reported more favorable data than they actually measured. This suggests that some patients might be inclined to select more favorable values for registration, which could leave early signs of graft failure unnoticed. Additionally, the majority of patients did not register their measured values on the day of measurement, but saved up measurements over several days to register them all at once. This so-called delayed registration was the main reason for patients not having followed up the advice to contact the hospital in case of a significantly increased level of creatinine.

This study is part of a larger project in which the safety and usability of self-monitoring kidney function after transplantation supported by an SMSS are investigated. Our results showing that patients seem inclined to select more favorable creatinine values for registration and to postpone registration suggest a challenge to the safety of self-monitoring. This should be well considered when designing self-monitoring care systems, for example by ensuring that self-measured data are transferred automatically to an SMSS. Using devices that can transfer data automatically and providing active feedback to patients (eg, by sending text messages or emails) instead of having patients to log on to a website will eliminate the issues of data selection and delayed registration, and as such contribute to the safety of self-monitoring kidney function after transplantation.

## Abbreviations

eGFR: estimated Glomerular Filtration Rate
IQ: interquartile range
LUMC: Leiden University Medical Center
MAP: mean arterial pressure
RCT: randomized controlled trial
SD: standard deviation
SMSS: Self-Management Support System
